# User Perspectives on Barriers and Facilitators to the Implementation of Electronic Health Records in Behavioral Hospitals: Qualitative Study

**DOI:** 10.2196/18764

**Published:** 2021-04-08

**Authors:** Se Young Jung, Hee Hwang, Keehyuck Lee, Donghyun Lee, Sooyoung Yoo, Kahyun Lim, Ho-Young Lee, Eric Kim

**Affiliations:** 1 Department of Digital Healthcare Seoul National University Bundang Hospital Seongnam Republic of Korea; 2 Signature Healthcare Services Los Angelis, CA United States

**Keywords:** electronic health records, mental health care, qualitative study, mobile phone

## Abstract

**Background:**

Despite the rapid adoption of electronic health records (EHRs) resulting from the reimbursement program of the US government, EHR adoption in behavioral hospitals is still slow, and there remains a lack of evidence regarding barriers and facilitators to the implementation of mental health care EHRs.

**Objective:**

The aim of this study is to analyze the experience of mental health professionals to explore the perceived barriers, facilitators, and critical ideas influencing the implementation and usability of a mental health care EHR.

**Methods:**

In this phenomenological qualitative study, we interviewed physicians, nurses, pharmacists, mental health clinicians, and administrative professionals separately at 4 behavioral hospitals in the United States. We conducted semistructured interviews (N=43) from behavioral hospitals involved in the adoption of the mental health care EHR. Purposeful sampling was used to maximize the diversity. Transcripts were coded and analyzed for emergent domains. An exploratory data analysis was conducted.

**Results:**

Content analyses revealed 7 barriers and 4 facilitators. The most important barriers to implementing the mental health care EHR were the low levels of computer proficiency among nurses, complexity of the system, alert fatigue, and resistance because of legacy systems. This led to poor usability, low acceptability, and distrust toward the system. The major facilitators to implementing the mental health care EHR were well-executed training programs, improved productivity, better quality of care, and the good usability of the mental health care EHR.

**Conclusions:**

Health care professionals expected to enhance their work productivity and interprofessional collaboration by introducing the mental health care EHR. Routine education for end users is an essential starting point for the successful implementation of mental health care EHR electronic decision support. When adopting the mental health care EHR, managers need to focus on common practices in behavioral hospitals, such as documenting structured data in their organizations and adopting a seamless workflow of mental health care into the system.

## Introduction

### Background

Mental illnesses are common and costly for patients and their families. It is estimated that in 2017, 970 million people worldwide had a psychiatric or substance use disorder. The largest number of people had an anxiety disorder, estimated at approximately 4% of the population [1]. More than half of Americans will have had a clinically significant mental illness, such as a psychotic disorder, a major depressive disorder, an anxiety disorder, or a substance use disorder [2]. People with mental illnesses also have higher rates of coexisting medical diseases, lower life expectancies, and health care costs that are 2-3 times those of people without mental illnesses [3]. If medical information is shared and managed between medical institutions, this problem can be effectively managed by reducing duplicate prescriptions and proactively detecting and intervening in psychiatric problems. Prerequisites for the exchange of medical information are the dissemination of electronic health records (EHRs).

The Health Information Technology for Economic and Clinical Health (HITECH) Act was prompted in 2009 by evidence that the use of EHR can improve the quality of care delivered [4]. HITECH drove annual increases in EHR adoption by 8% points after implementation of the act’s incentive program [5,6]. However, EHR use in mental health settings is still lagging behind. Compared with general medicine and surgical hospitals possessing certified EHRs, only 49% of psychiatric hospitals have certified EHRs as of 2017. The number is lower than rehabilitation (89%), children’s (87%), and acute long-term care (59%) hospitals [6,7].

### Previous Studies

Previous studies have suggested that the use of EHRs has greatly impacted the ways in which health care professionals document and manage patient information [8-11]. Functions in mental health care EHRs as well as their provision in hospitals are important. In fact, previous studies revealed that 27.7% of patients with bipolar disorder and 27.3% of patients with depression lacked a diagnosis of their mental illness in their primary care EHRs. Furthermore, data on mental health patient-provider encounters occurring in nonprimary care settings were often completely absent from the primary care record [12]. A previous study suggested that 3 prominent barriers to the adoption of mental health care EHRs should be considered: complex privacy-related laws and regulations, inadequate financial incentives, and assistance for psychiatric providers [2]. Another study suggested that EHRs designed for mental health care must satisfy the needs of clinicians for the successful uptake of necessary information and improvement in patient experience to disseminate them efficiently [13]. Although a number of studies have evaluated the effectiveness of EHRs, there have been few studies on barriers and facilitators to the adoption of mental health care EHRs from the perspective of mental health clinicians, nurses, pharmacists, and administrative professionals in mental hospitals.

### Aim

The objective of this study is to analyze barriers and facilitators to the adoption of mental health care EHR. To address them, we thoroughly interviewed end users with a qualitative research method when they make transition from paper medical documents to EHRs.

## Methods

### Design

In this study, we used phenomenology. It is an approach to qualitative study that focuses on the commonality of an experience within a participating group. Through a phenomenological approach, we aim to construct a universal meaning of experience. By conducting a qualitative analysis based on semistructured interviews with mental health clinicians and administrative professionals, we sought to explore barriers, facilitators, and ideas regarding the implementation and usability of a mental health care EHR. This study followed the Consolidated Criteria for Reporting Qualitative Research Guidelines (Multimedia Appendix 1 [14]).

### Setting and Participants

This study was conducted at 4 behavioral hospitals under the same parent corporation. The hospitals provide both inpatient and outpatient services for mental health and substance abuse treatments in 2 states.

In 2017, corporate leaders decided to implement an EHR. Owing to the lack of sophisticated behavior-oriented EHR systems, they launched a project to customize an advanced medical EHR into a behavioral version by tailoring it to the workflow of behavioral hospitals. The long-term goal was to adopt this mental health care EHR, called BESTCare 2.0B, across all the behavioral hospital branches after successfully implementing a standardized mental health care EHR in the first hospital [15-17].

The pilot project was initiated at a behavioral hospital in California. The hospital was selected because it was the most acute hospital with more complicated workflows than its sister hospitals. A group of clinicians and business analysts from the vendor underwent a 2-month on-site gap analysis. They then worked with information technology engineers for 6 months to customize a general hospital’s EHR into a behavior-specific version.

One of our researchers, DL, is a business analyst who participated in developing a standardized version of the mental health care EHR for the Aurora hospitals. In the pilot project, he analyzed the paper-based workflow by determining requirements, helped to design the To-Be process, and suggested alternatives. He also trained physicians, nurses, pharmacists, mental health clinicians, and administrative professionals before the implementation; supported the go-live process; and followed up with modification requests from 2 hospitals located in California. With his extensive experience in the mental health care EHR implementation project and with the help of other trainers, the study participants were selected through purposive sampling. We concluded candidates to be suitable for the interview based on the following criteria: (1) must be an active user of the mental health care EHR; (2) is identified as a representative of their job group in having a profound understanding and is responsible for knowledge management inside the department; or (3) must be at a management level and must be well acquainted with the electronic process with the mental health care EHR. Then we contacted the CEO and directors to schedule the interview with the identified candidates. However, as some of the candidates were unavailable during the interview sessions, we asked the directors to recommend a substitute who best matched the aforementioned criteria. Therefore, this study had originally planned purposive sampling but conducted snowball sampling to find the rest of the study participants. As for the quota, we mainly focused on studying users with a clinical background, such as physicians, nurses, pharmacists, and mental health professionals. The study only included a few administrative professionals who best represented their job group, as stated in the criteria. As many physicians from hospital A submitted requirements, suggestions, and feedback while developing the standardized version of the mental health care EHR, the largest number of physicians were interviewed as they best understood the system and its implementation. Hospital B was relatively smaller in size than hospitals A, C, and D and had fewer staff to interview. However, because of fewer human resources, administrative departments such as Health Information Management (HIM) were much more in need of using and maximizing the benefits of the mental health care EHR. They were recommended for the interview by others as acknowledged to be thoroughly aware of how the system works. Only one pharmacy director ran the inpatient pharmacy in all 4 hospitals, and they were all interviewed. The director of clinical services in hospital A had retired and was unavailable.

One of the criteria for purposefully selecting our research participants was that the person had to be identified as a representative of their job group in terms of having a profound understanding and should be responsible for knowledge management inside the department or had to be at a director level and well acquainted with the electronic process. To identify these participants, we collected opinions from mental health care EHR trainers who also supported each department at the time of go-live process. In addition, we had the director from each department confirm that they are suitable for the interview when asking to schedule the time. However, in terms of gender ratio, age distribution, and experience of EHRs, there were apparent limitations because when considering these aspects, not all participants fit into the aforementioned criteria. For example, all physician participants who met the criteria were male. Of the 20 nurses, 15 were female and 5 were male. More than half of them were in the age group between 31 and 40 years. There was only one pharmacy director in each hospital, and all were female. In addition, the 4 hospitals could not fully represent mental health hospitals. They are located in only 2 states—2 in California and 2 in Arizona. At a minimum, state law, clinical practice, and work patterns can be different from one state to another, requiring at least more than one hospital in each state to represent mental health hospitals in the United States.

The interview was conducted at 4 of the 5 behavioral hospitals where the mental health care EHR was implemented. A semistructured interview questionnaire was formed through biweekly meetings until every researcher agreed on the contents of the questionnaire (Textbox 1).

**Semistructured interview questionnaire.**

**Interview questions**

- What was your first impression of the mental health care electronic health record (mental health care EHR) implemented in this hospital?
- Did you have experience with other behavioral or general electronic health records before?
- How long did it take for you to get comfortable with the mental health care EHR?
- Were there any barriers to the implementation of the mental health care EHR?
- What do you think would have helped to better implement the mental health care EHR?
- What screens or functions do you use in the mental health care EHR and how do they impact your daily practice?
- Do you think the communication has gotten better or worse with the mental health care EHR?
- How is the navigation when using the mental health care EHR?
- What do you think of the Clinical Decision Support System in the mental health care EHR?
- What are your thoughts on using tablet PCs in the inpatient unit? How would you like to utilize tablet PCs on the floor?
- Are there any issues with the mental health care EHR that need to be resolved?
- Do you think the mental health care EHR is well customized for the behavioral workflow? Is there anything missing?
- What functions or features do you want to add to the mental health care EHR?
- Do you have any suggestions or recommendations for the mental health care EHR to improve your work experience?
- Is there anything else you want to mention regarding the mental health care EHR?

After a thorough review of the data by 2 experienced qualitative investigators, we created a preliminary codebook, with separate codes for patient and physician transcripts. Using inductive content analysis, the authors coded all transcripts with additional steps to ensure validity. We addressed discrepancies and reached a consensus in biweekly meetings. Throughout the coding process, the team discussed and revised the codebook and returned to the previously analyzed transcripts to ensure consistency.

As the mental health care EHR was launched at the hospitals in different time periods, the hospital’s length of experience in using the mental health care EHR varied at the time of the interview, as presented in Table S1 of Multimedia Appendix 2. Before the adoption of the mental health care EHR, 2 hospitals located in California had been using paper, except for a stand-alone electronic pharmacy management system for the pharmacist to enter paper-signed medication orders. In contrast, physicians, pharmacists, and nurses from 2 hospitals in Arizona had been using a legacy computerized physician order entry (CPOE) system. The characteristics of participating hospitals are presented in Table S1 of Multimedia Appendix 2.

The study participants were selected through purposive sampling [18]. We aim to include participants from various professions who had in-depth knowledge of the work process with the mental health care EHR. Potential quotas for purposive sampling were more than 20 people for doctors and nurses and more than 3 people each for administrative positions and pharmacists to listen to the opinions of direct users of the mental health care EHR and those of support positions.

Several participants were recommended by hospital management. Most of them were generally acknowledged as *Superusers*, whom their colleagues would look for if they initially ran into an issue with the mental health care EHR, implying that they had a good understanding of the operation of the mental health care EHR in day-to-day tasks.

### Data Collection

DL and KL conducted face-to-face, semistructured interviews. DL, a male researcher with a BA degree who had been working in the pilot project in developing the mental health care EHR as a business analyst, led the interview. He had also trained physicians, nurses, pharmacists, and mental health clinicians before the implementation of the mental health care EHR in 2 Aurora hospitals located in California. The positive relationship with the study participants in the 2 hospitals helped to create an open atmosphere, thereby encouraging them to speak more at ease about their experience. A female researcher with an MS degree, KL, who had no previous relationship with the study participants, took notes during the interviews. Both interviewers received training for qualitative interviews. Interviews were audio recorded in a closed office or conference room with a recording device and lasted from 20 to 60 minutes. Nobody was present in addition to the participants and researchers. During the sessions, DL followed a semistructured interview questionnaire that covered topics related to their experiences and thoughts regarding the mental health care EHR. Although most of the sessions were conducted one-on-one, 4 sessions were interviewed in a group of 2 and 1 session was interviewed in a group of 3 because of working shifts and time limitations. The questions for the group interviews were identical to those of the one-to-one interviews. The researchers adopted the interview guidelines that were developed based on previous research and approved by members of the eHealth research team at Seoul National University Bundang Hospital (SNUBH).

### Data Analysis

We used an explorative content analysis method to capture the perspectives concerning the barriers and facilitators when adopting an mental health care EHR. The recorded interviews were transcribed by an external professional transcription service and were further repeatedly reviewed and corrected by a researcher (DL) to enhance the accuracy of the transcriptions. All anonymized transcriptions were uploaded to Dedoose software and coded for analysis. To ensure reliability, the transcripts were independently read and coded by 2 individual researchers (DL and KL). The initial codes were inductively generated from the data. The codes were grouped into 3 major themes: barriers, facilitators, and suggestions or ideas by each profession. The identified barriers and facilitators relevant to the study were discussed regularly until both researchers agreed that they had reached saturation. All the researchers verified the results until we reached a consensus on the clarified themes.

### Ethics

Before the interviews, the researchers explained the research objectives and purpose of the study. Consent forms were signed by all participants voluntarily. None of the participants refused the interviews. This study was approved by the Institutional Review Board of Human Research of SNUBH, Republic of Korea (Protocol No. B-1904-534-301).

## Results

### Participant Demographics

We conducted interviews between October 31, 2020, and November 19, 2020. A total of 10 physicians, 20 nurses, 4 pharmacists, 5 mental health clinicians, and 4 administrative professionals participated in the interviews. Many management-level personnel such as medical directors, chief nursing officers, directors of each department, and managers or supervisors also participated. From a total of 43 participants, the study included 19 directors, 10 managers or supervisors, and 14 end users, as presented in Table 1.

**Table 1:** Demographic characteristics of participants (N=43).

Category and variables			Value, n (%)
**User group**			
	Physicians	10 (23)	
	Nurses	20 (47)	
	Pharmacists	4 (9)	
	Mental health clinicians	5 (12)	
	Administrative professionals	4 (9)	
**Gender**			
	Male	18 (42)	
	Female	25 (58)	
**Age (years)**			
	<30	1 (2)	
	31-40	18 (42)	
	41-50	8 (19)	
	51-60	9 (21)	
	61-70	6 (14)	
	≥70	1 (2)	
**Career (years)**			
	<9	15 (35)	
	10-19	12 (28)	
	20-29	10 (23)	
	30-39	4 (9)	
	≥40	2 (5)	
**Number of EHRs^a^ used before**			
	0	9 (21)	
	1	7 (16)	
	2	11 (26)	
	≥3	16 (37)	
**Management level**			
	Director	19 (44)	
	Manager or supervisor	10 (23)	
	End user	14 (33)	
**Facility**			
	Hospital A	13 (30)	
	Hospital B	9 (21)	
	Hospital C	11 (26)	
	Hospital D	10 (23)	

^a^EHR: electronic health record.

A total of 10 physicians included 8 psychiatrists and 2 internists. Nurses were working in various locations and roles, including inpatient ward nurses, medication nurses, assessment and referral nurses, education nurses, and utilization review nurses. In all 4 hospitals, only 1 pharmacy director operated the inpatient pharmacy during the day with one or more pharmacy technicians. Therefore, we interviewed all the 4 pharmacy directors. Mental health clinicians included licensed therapists, counselors, and social workers. Administrative professionals included HIM professionals, compliance officers, and business development officers.

### Barriers

**Figure 1:**
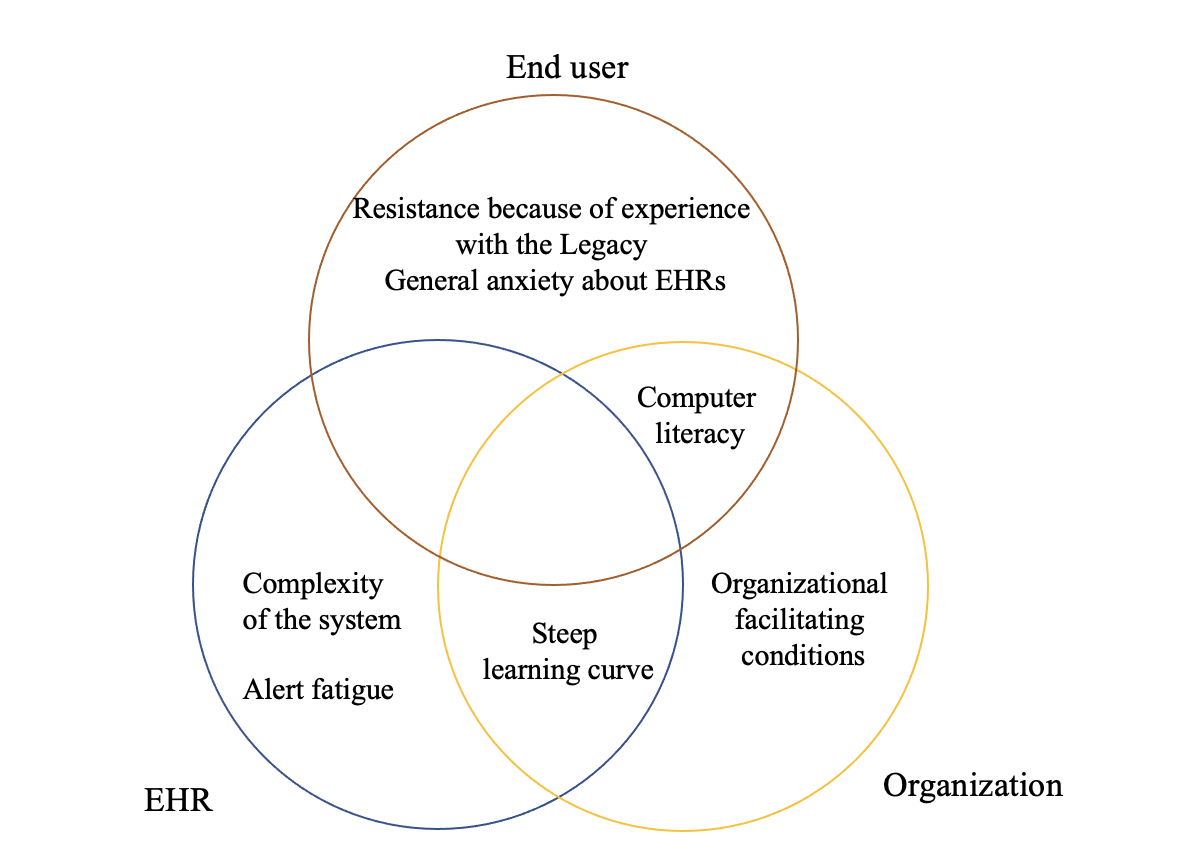
Parties or elements requiring improvement to overcome the 7 barriers. EHR: electronic health record.

We found 7 major barriers to benefitting from the mental health care EHR (Figure 1).

#### Computer Literacy

The skill sets to handle tasks with an EHR varied by profession. Nurses most frequently reported that their colleagues encountered difficulties with computer proficiency. A number of them were simply not exposed to computers in the past. The lowest level of computer literacy was having trouble with double-clicking the mouse. Although the mental health care EHRs required single clicks most of the time, failing to double-click disrupted nurses to proceed to the next step. The next hurdle for nurses familiar with controlling the mouse and keyboard was not being able to manipulate multiple screens and functions according to their needs:

We have some nurses that aren't really used to working on computers. They didn't have to before for this job really. That was the biggest challenge.

B022, Nurse

Physicians mostly mentioned having trouble with slow typing, because of the relatively lengthy psychiatric assessments and narratives that summarize the patient’s diagnosis and condition. Almost all of these participants self-referred themselves as being old.

#### General Anxiety Toward EHRs Among Staff

Not only did the staff with inadequate computer skills physically have trouble with the EHR but they also had psychological pressure in the process. The spread of anxiety in using the mental health care EHR had been affecting some of the older staff to consider quitting when perceiving the system as too modern for themselves:

The first day I came, I thought maybe I'm going to put out my resignation letter. I didn't know where this was leading me to.

B031, Nurse

All physicians who were trying the mental health care EHR for the first time reported that they did not have a good first impression of the system. This became a mental barrier to accepting the new technology:

It came across as very detailed, but complicated and overwhelming. Especially for somebody like me who is not EHR experienced. I haven't had an EHR before. No, I have no experience with other EHRs before this one.

C001, Physician

#### Complexity of Mental Health Care EHR

The layout of the user interface (UI), including the alignment of patient information, orders, medical records, assessments, exam results, and a bird’s-eye view of the patient’s treatment history, had often been reported to be complicated at first. The majority of physicians who had not been using an EHR before provided negative comments about the perceived complexity and ease of use:

The first impression, honestly, was that it's going to be too much work and that it's not that easy to use.

C002, Physician

Many nurses, if not overwhelmed by their low computer literacy, have reported difficulty with the mental health care EHR in finding specifics on many different screens. The mental health care EHR had set its core features in each specialized screen and allowed multiple ways to access them to accommodate preferences of each user. That, in the beginning, confused the nurses, as they had the broadest range of access to screens from the nursing module, multidisciplinary care plans, and even order-related screens:

For the nursing piece, there's quite a bit to navigate through finding the medical care plans, finding the “Order Entry.” The navigating for nursing is by far the hardest in the building and the most in-depth.

B026, Nurse

Pharmacists generally thought that information displayed to them was too detailed. They reported that it was not a problem for them clinically, but the barrier was more related to the pharmacy system being too big for its size and resources available in a small inpatient pharmacy:

If you're in a larger hospital where there's a lot of staff, this is appropriate. But where we don't have as much staff, I think, and we're a smaller hospital, I think all of the different features, it's a little too much and overwhelming for everyone.

B027, Pharmacist

#### Steep Learning Curve

Among the mental health professions, the majority of the inpatient nurses perceived that there was a lot to understand to get used to the routine tasks conducted in their stations through the mental health care EHR:

It's just that, to learn so many new things all at once was the hard part, I think.

B006, Nurse

On the contrary, mental health clinicians and administrative professionals mentioned far less difficulty in overcoming the learning curve.

#### Alert Fatigue

Some pharmacists complained of the excessive number of alerts from the Clinical Decision Support System (CDSS) when verifying medication orders. Although these alerts were categorized into multiple levels, which required different actions such as selecting a reason for overriding if it was a high-level alert and having to read and pass the informative message if it was minor, some reported the fatigue to be accumulating regardless:

What came up was “high dose over recommended max,” but there's so many alerts that I feel as if everyone's going through decision fatigue, because everyone's just like, “bypass, bypass.”

B010, Pharmacist

Inpatient nurses had a similar issue with *New order alert* notifications. This feature refers to nurses receiving alerts regarding any new or updated orders from physicians issued to patients in their inpatient ward. Although this feature provided options to control the frequency of alert notifications by oneself, some nurses had turned this off because of alert fatigue:

They’re constantly getting the [New order] alerts and sometimes the nurses will turn that feature off to not have to keep getting those alerts.

B017, Nurse

Knowing the circumstances, some nurse managers made requests to modify the system by turning on the nurse’s alert notification by default.

#### Organizational Facilitating Condition

The corporate central medical committee has been managing the medical contents and regulatory compliance of the 4 hospitals. Notably, it played a vital role in the adoption of the mental health care EHR by standardizing the electronic forms and their contents. Although the participants appreciated the overall facilitating condition, physicians mentioned that clinical materials needed correction even after the implementation:

I think it definitely is a robust system. The thing is that a lot of it is our own doing. I don't think the [mental health care EHR] had anything to do with it. Forms we asked are added as we told them, but some of them need to change. For example, there is an element called “patient insight.” All the choices are related to poor insight, so you have to use “Other” and then write it. “That patient has fairly good insight.”

C009, Physician

In addition, some participants pointed out that there were technical support and communication channels to the corporate committee. However, organizational decision making should have been more agile after adoption:

And so the channel is there, but in the process when we all launched this, the changes were very slow. ...the impression I got is that it was very slowly implemented at the time.

C002, Physician

The fact that the corporate committee did not follow up and provide feedback for the modification requests swiftly as expected by end users seemed to negatively impact even those who were satisfied with the successful adoption of the mental health care EHR:

I think the initial implementation was very good. Very good. The follow-up, not so much. As I go along and it is very difficult to communicate, to who is the one who is going to address it and what is the priority? ...I think we make a request to the hospital identified person and we don't know then what happens.

C009, Physician

#### Resistance Because of Experience With the Legacy System

As mentioned earlier, unlike the hospitals in California, 2 hospitals in Arizona had been using a legacy CPOE system. Experiences of pharmacists and nurses with the legacy system had affected the perceived usability of the newly adopted mental health care EHR. The resistance from a couple of pharmacists was because of the idea that one screen was sufficient for the task, as compared with 3 separate screens full of information:

Our old system, everything was on one screen. The doctors were entering orders on one screen. We were verifying on the same screen. It was all done on the same screen. There's three screens now. Whereas before, everything was just done on one screen. It wasn't all of this information.

B020, Pharmacist

They also previously performed the Closed-Loop Medication Administration (CLMA) with a barcode scanner. CLMA, which greatly reduces medication errors by checking the 5 rights, was no longer available with the mental health care EHR. The absence of a legacy system’s drug administration scanning functionality caused medication nurses to feel ill at ease during the administration process. While acknowledging other benefits and strengths of the mental health care EHR, nurses were asked to add back the safety net that was in place:

We had the scanning features. We had the [CLMA] alerts. It's really just fine tuning so that they feel comfortable with it.

B026, Nurse

### Facilitators

**Figure 2:**
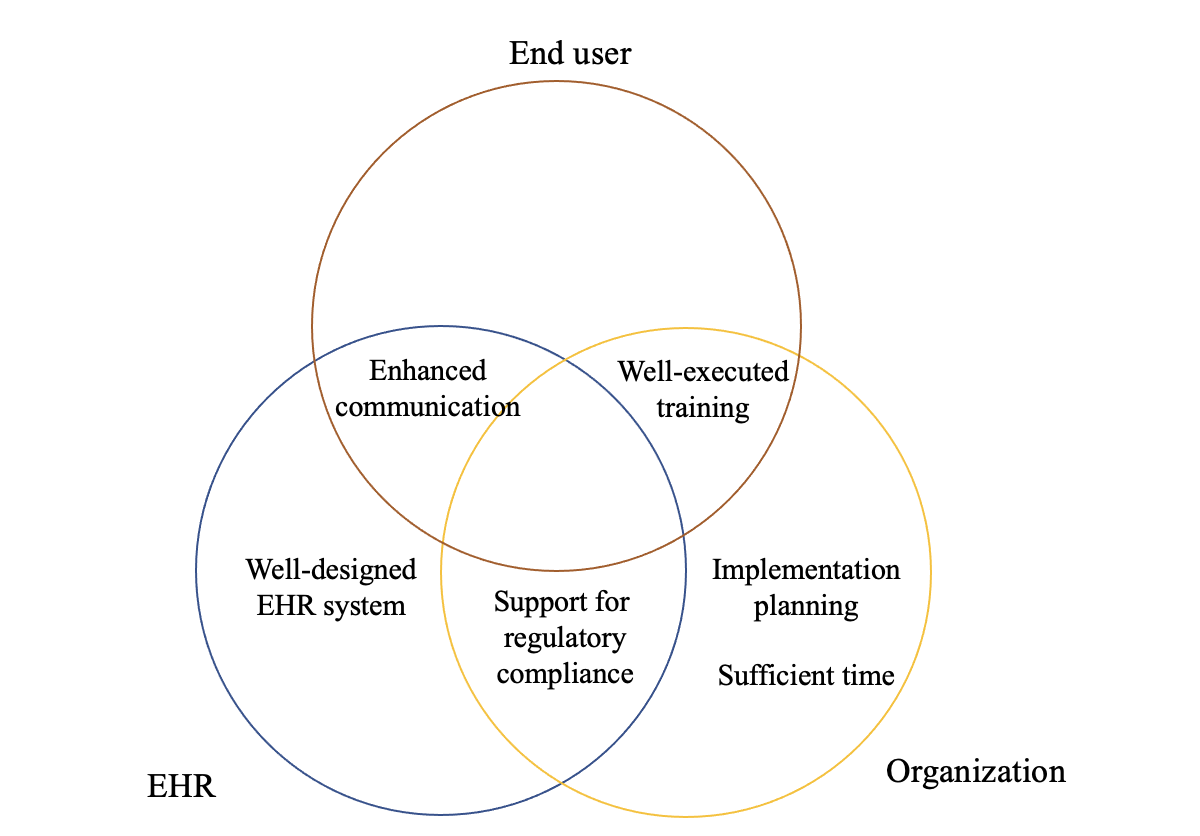
Parties or elements related to facilitators. EHR: electronic health record.

We found 4 significant facilitators to benefitting from mental health care EHRs (Figure 2; Textbox 2).

**Facilitators to benefitting from the mental health care electronic health record.**

**Mental health–centered electronic health record (EHR)**

- EHR tailored to behavioral workflow
- Support multidisciplinary documentations
- Support regulatory compliance

**Well-designed EHR system**

- Easy to use (perceived ease of use)
- Improved productivity
- Good usability
- Flexibility of forms

**Advantages of using an EHR**

- Easy access to patient information
- Improved patient safety and quality of care
- Enhanced interprofessional communication

**Implementation strategy**

- Well-executed training
- Sufficient implementation time
- Implementation planning

#### Mental Health–Centered EHR

In terms of accommodating its unique workflow, physicians who had experience with other EHR systems addressed the fact that the system accomplished that minimum but crucial requirement and showed gratitude for not being a workaround within a general EHR:

This mental health care EHR is more comprehensive and more relevant to psychiatry. With other EHRs, relevance to psychiatry is not high. They have a little bit of a section of psychiatry. This is more relevant, and I find it comprehensive, easy to use, and navigate it very well.

C001, Physician

Nurses and mental health clinicians echoed the same thoughts. From nurses working in the intake department who first assessed and admitted the patient to the nurses working in the inpatient unit, all had agreed that the work process was well integrated into the mental health care EHR:

This mental health care EHR is drastically different. I think it fits well for what we do, workflow wise. I have no issues using it, training on it.

B001, Nurse

Another element that differentiates mental health from other specialties is the multidisciplinary treatment team working together to provide patient care. The team discusses the patient's problems and collectively sets up treatment plans and documents parts of the sections defined by the job group. The mental health care EHR supported multidisciplinary documentation, and this was a great relief to mental health professionals:

All the clinical forms and everything that we need to do, treatment plans, all that, it flows nicely.

B012, Mental health clinician

In addition, administrative professionals and directors felt that the mental health care EHR contributed in ensuring that the regulatory requirements were in place. Regulatory compliance had always been a delicate issue when working on paper. However, it proved to be less of a burden when the mental health care EHR managed to satisfy the surveyors with what was mandated:

I'm in charge of all the joint commission and Center for Medicare and Medicaid surveys, and the mental health care EHR has everything in there that a surveyor would want to see. I got to give it to them, they did a good job form-wise, to make sure everything was there.

B013, Administrative professional

#### Well-Designed EHR System

In addition to developing an EHR to run in the behavioral setting, it was equally crucial for the mental health care EHR to deliver the advantages of using a thoroughly designed electronic system. Despite having a high number of negative first impressions from all professions, many felt that the mental health care EHR was easy to learn during the training sessions. Most physicians found that this system was not difficult at all:

Ordering is pretty self-explanatory. Overall it's well presented. I think presentation-wise, it is clear. I think initially there were a lot of tabs that made some sort of concern on how many tabs there were, to kind of get adjusted. But after the first training session it actually made sense and it felt organized in a categorical way.

C004, Physician

Although numerous nurses had to break out of their comfort zone in trying out modern technology, they started to gain confidence in a short time period:

And when I first started to see it, it became clear to me that it was going to be pretty easy. So very user friendly and easy to follow. I liked it. I made the correlation between a smartphone with the apps. So, my brain got it.

B001, Nurse

Superusers shared their thoughts of the mental health care EHR with their colleagues when training for go-live process. Positive opinions on the perceived ease of use encouraged a vast majority of the nurses to see what they could take advantage of every day:

I tell people this when I'm training them, if you can use your smartphone, you can use the mental health care EHR. It's that simple. I tell people it's one of the easiest systems I have used. So that would make it in itself one of the best systems. And I have had a few nurses that have worked at other hospitals and they too have said that this is one of the easiest systems that they have used. Overall it's very easy to follow. It's very easy to figure out and you can solve it on your own. So, yes, it's definitely efficient, effective, and easy.

B001, Nurse

To mental health clinicians who facilitated group sessions for patients, using the mental health care EHR meant spending much less mental and physical effort throughout the day:

After it launched, I was very excited. It was quick and fast. Going from printing out over 200 documents a day and using over 200 labels a day, running out of pen ink, hand cramps. So I was excited that it was electronic because things were done smoother.

B021, Mental health clinician

In addition, mental health clinicians highlighted efficiency, the time-saving merit of the system, in completing electronic attendance and daily documentation tasks within the time frame:

What we have now is efficient and it's cut a lot of time out because we used to have to sign all the notes then go and put them all in the charts. That was terrible. It just took forever.

B033, Mental health clinician

Participants also emphasized that the multitaskable UI had greatly helped to access additional information without disruption of the main task in hand:

Navigation-wise it's good. I like that you can view two screens at the same time, because that really helps. If I'm talking to a patient about meds, I can have my documentation open, but also looking at the meds. We like that and a lot of the nurses like that. Being able to close an item, open another item quickly, rather than some EHRs, where you have to document this and to move on, you have to document here. We're able to jump around and move.

B005, Nurse

Another characteristic of the mental health care EHR is the support of personalization options to accommodate end users’ preference according to their work patterns. Many nurses and mental health clinicians indicated the customizable *My Favorite menu* to be helpful in setting the tasks and documentation in a certain order they can simply follow in sequence:

The favorite menu for me is my favorite. Because I can really just save the documents that I regularly use and search from there. The quick mental health care EHR shortcuts. Those shortcuts make a huge, huge difference.

B021, Mental health clinician

This personalization feature was beneficial for nurses who had trouble remembering where to go for their routine work. Having the favorites set up to all end users, especially to nursing staff, before go-live process became a priority that ensured a smoother transition:

All the nursing staff, we tried to help them set up their favorites and all that so it would be ready to go.

B013, Administrative professional

The flexibility of the mental health care EHR had also been reported as a relief to the administrative professionals. Knowing that the *Form generator* can add or edit documentation templates in an instant made it easy for the staff to accept the new electronic system:

Usually when we have a problem and you get it done pretty quick, and no EHR is perfect. I know that. But as long as when we need something changed, it's fairly easy to change it. I know I've asked (Corporate coordinator) a couple times just to switch a couple things around and he can do it himself, which is nice.

B013, Administrative professional

#### Advantages of Using an EHR

Participants were delighted to enjoy the universal benefits of using an EHR. A common theme among participants was improved access to patient information. With the help of the mental health care EHR, the difficulty in finding physical patient charts, which was delayed if occupied by another staff member, and, therefore, not being able to access patient information promptly had been resolved:

It actually makes my job so much easier because I can access the information from whatever location I'm at, as opposed to going to the units and pulling out the charts. And it's easier for me to get the information at the same time someone's looking into the chart, because otherwise I have to wait to see when it's available.

B006, Nurse

Before, administrative professionals who did not even work in inpatient or outpatient units had greater difficulty in locating patients’ charts. Finally, they were able to access patient information in time through the mental health care EHR:

This mental health care EHR has really helped me because it used to be I could never get information. I'd have to go pull the patient's chart, I'd have to go to the unit, I'd have to go to Medical Records and try. So it wasn't until we got the mental health care EHR that I could even look this up. Before, we weren't capturing that. It was captured on the paper chart, but I couldn't find it easily at all.

B007, Administrative professional

Participants believed that the mental health care EHR had improved patient safety and quality of care provided to the patients. Physicians and nurses were pleased that the mental health care EHR’s CDSS alerts helped them to be less prone to making medication errors compared with writing orders in paper and sending it to the pharmacy:

It [CDSS] gives that extra layer of protection for the patient.’

C004, Physician

Especially with the alerts just for medication alone. Because before on paper, we'd have to wait anywhere from 20 to maybe two, three hours when pharmacy would get the information, enter it and realize, Oh, that patient has an allergy or these two medications are contraindicated. That system tells you right then and there. So that's helpful.

B001, Nurse

They reported that any mistakes made were caught instantly by other clinicians, not to mention that they did not have to decipher handwriting anymore:

Medication errors are definitely going down. In this way, everything is in print. Everything is concrete so that if it is a mistake, if the doctor's ordering in different ways, the pharmacist and nurse can visualize and then say, “Hey, this is the wrong way,” communicate with the physician because they can see the order very clearly. It definitely improves the patient's safety.

C005, Physician

Participants were very satisfied with the enhanced interprofessional communication. As mental health involves collaboration from various clinicians, participants felt that better communication between the members of the treatment team empowered to work together toward the established goals:

I think communication is better with the mental health care EHR because there is continuity of care, communication amongst the team, the different disciplines. I think the mental health care EHR has really put us to another level unlike before. I can see if I'm working on that patient and the counselor is working on that patient, I'm able to read the note what the patient did in the group. So I think it's really amazing.

B019, Nurse

Mental health clinicians were also impressed that they began to receive feedback from other clinicians when using the mental health care EHR. They were positive toward the mental health care EHR in creating a sense that they are actually working collectively as a team, reflecting the work of their colleagues. This was less likely to be observed with paper charts:

As far as communication as a whole, I found that we've had social workers come up to us in our department, “Wow, your notes are so detailed,” and “Oh, your notes are really descriptive with your team,” and it's great that they're really reviewing the chart and taking time to read other's documentation.

B021, Mental health clinician

#### Implementation Strategy

All participants considered training to be the most influential factor in getting used to the mental health care EHR and ready for transition. A couple of physicians who were aged more than 70 years at the time of implementation stated that good training sessions had changed their attitude positively toward the adoption of mental health care EHR:

The first training experience was actually very, very good. I felt very comfortable using the system.

C004, Physician

Many nurses and mental health clinicians were also pleased with the thorough training sessions and continuous support from trainers. Training sessions were scheduled by each department for a maximum of 15 users in one session with 2 or 3 trainers. Instructions were provided by their work patterns with real-life scenarios set up in the training environment of the mental health care EHR. All users had to pass the competency test at the end of the training session for what they had learned. By observing the users going through the competency test during the session, trainers were able to easily identify the additional guide they needed:

I thought that the rollout was really good. I think it was very thorough in teaching all of us what we needed to know.

B012, Mental health clinician

Additional training was provided to staff who needed more help. The training room was open for practice to welcome those who needed extra hours and one-on-one training:

I think the people that came out and trained us last time were really helpful. I really liked them. I felt like they were always there when we needed them. So the training was for, in my opinion, really, really good. I don't think that that could have been much better.

B032, Nurse

The trainers not only guided them with well-prepared contents and extra training sessions but also put much effort into encouraging and boosting morality. Many end users were known to have gained confidence in overcoming the mental barrier at this time:

When I attended the third time, I met one of the senior trainers. So I called him, I said, “You know what? This, your (mental health care EHR) is going to send me out of job.” He started laughing. He said, “[Name], I know you can do it.” ...And he started showing me. That was the end. The third time, I got it. The first time, I was totally lost.

B032, Nurse

Another facilitator that was frequently mentioned by participants was having enough time for accepting the mental health care EHR. The implementation support team meticulously planned the initial transition from paper to the mental health care EHR during the go-live process and the implementation process, keeping up with the project schedule. Users had a minimum of 2 months for preparation, even after the customization of the mental health care EHR. Even physicians mentioned that the given time was sufficient:

I think they have been very kind and they have been very patient. And that was very helpful. Compared to the other hospitals where I work, we didn't have this much support. And they have taken a good amount of time for the implementation.

C005, Physician

When asking the participants what would help the remaining sister hospitals in adopting the mental health care EHR, nurses also stressed that giving time for transition and training is critical:

I would have a recommendation, maybe some more time, which they did give us. I think the trainers we had really did a good job. They gave us time, they weren't like hurrying us, no. So I would put emphasis on that.

B019, Nurse

According to some participants, one of the most effective implementation plans was to use the forms in the mental health care EHR even before the go-live process. This way, everyone was already well acquainted with the contents of what was to come, and when using the electronic version, at least the forms were the same and much easier. They felt that the benefits of the mental health care EHR were easily recognized in this aspect:

So it's almost exactly what we were doing on paper. Just a computer form, quicker, better, nicer.

B001, Nurse

### Ideas

#### Alerts to Address the Legal Status

In behavioral hospitals, psychiatric patients are often admitted with an emergency hold in which the law permits the facility to process an involuntary admission in certain circumstances. Although the duration for hold varies by state, all states have emergency hold laws, and mental health professionals need to address the duration before the permitted hold ends. Nurses were very eager to have a stronger level of alert, even hard stops, to notify physicians and nurses for further action. In their opinion, addressing hold was the top priority and simply showing the current status and remaining hours was not enough to draw attention:

Maybe by suspending some kind of function to where it's not going to let you move forward until this is addressed. A pop up screen that offers, “not addressed” or “addressed.” So, it can change the legal status to show that the user has not taken care of this order yet. If it was not addressed, let's say by either discipline, doctor or nurse, hold ended one minute ago, boom, a pop up should appear. Showing it that the user needs to obtain legal status or order needs to be written. It's day two now and it still says pending, because somebody didn't address it. I think that would be helpful.

B013, Administrative professional

#### Autorecommendation of Laboratory Exams for Antipsychotics

Some pharmacists suggested that the mental health care EHR would be more helpful if the laboratory exams were paired with antipsychotics to be autorecommended when a physician issues orders. As they pointed out, one of the main objectives of an electronic system is to assist clinicians in removing burdens and to reduce human errors. Although the mental health care EHR was reported to be robust in handling complicated orders and protocols, they also wanted additional, automated support and guidance:

So when you have certain medications that are ordered, certain antipsychotics, I would think it would be helpful if ones would pop up and then the doctor could select, “Depakote, liver enzyme test” where they don't have to actually remember it and it just pops up, and then they just select which ones.

B010, Pharmacist

#### Use of Tablet PCs

To most of the participants, a well-designed mental health care EHR was satisfying in many ways. However, its limitation was clear. Users cannot carry it around and access it instantly. Although hospitals had laptops throughout the facility in some consultation rooms, medication rooms, and offices, many reported that the use of tablet PCs would be much more beneficial in certain areas. In 2 hospitals, nurses working in the intake department did not use laptops for assessment. They reported that entering results into the mental health care EHR via a computer after the assessment was quicker than the paper process; however, they felt that tablet PCs would make it easier to complete the intake assessment and receive electronic medical consent in the consultation room. They did not mind entering one long narrative later at the end:

I think tablets would be good in the Intake rooms when we're doing all the consents for the meds, because we have them just hand signing everything. I think it actually would be great in the Intake. I think that would speed things up and help out. Absolutely, it would. 100%.

B029, Nurse

Mental health clinicians shared the same thoughts in using tablet PCs to complete group notes during the group session. As of now, they were jotting down notes on paper to later recall and complete the notes on the computer:

If the tablet had (the bEHR) that I could go to them for the assessment part, that would make it easier. Because then I could tell them I'm just going to do an assessment real quick and check the boxes.

B033, Mental health clinician

Some nurses thought the round sheet was a perfect example of making use of a tablet PC. They believed forms that consisted of simple markings, the selection of drop-down menus, and only a few notes were good candidates:

Because every 15 minutes we have a sheet of paper that has a thousand check boxes and you check where they were, what they were doing. Every 15 minutes. Someday if we can come up with a form and have it all on a tablet.

B013, Administrative professional

Physicians also mentioned the round sheet, based on the idea that the few forms remaining in paper can switch to electronic forms by using tablet PCs:

I think if we had a tablet and the mental health worker doing the rounds does it, it's entered in the system right away versus you're writing it on paper and then having to put it in later. So I think it's much more efficient and less chance for errors to do it with a tablet right there.

C006, Physician

## Discussion

### Principal Findings

In this study, we found 7 major barriers and 4 major facilitators when implementing a mental health care EHR in behavioral hospitals. Of the 7 barriers, 3 are related to ease of use: computer literacy, complexity of the system, and steep learning curve. Other barriers include alert fatigue, poor organizational support, and general anxiety about the new environment. Of the 4 facilitators, 2 are related to the system itself: mental health–centered and well-designed mental health care EHR. One of the main facilitators is related to expectations from system use: improved communication, improved productivity, and better patient care. Organizational support relevant to implementation strategies is also an important facilitator. To the best of our knowledge, this is the first study to analyze the barriers and facilitators that are important for the introduction of mental health and EHR using qualitative methodology.

### Overlapping Characteristics of Barriers and Facilitators

An interesting finding is that the barriers and facilitators share overlapping characteristics. For example, some participants were worried about organizational support. However, some of them were satisfied with the support, such as good training and well-executed implementation planning. This means that a well-organized implementation plan and an educational support of an institution is one of the crucial elements for successful adoption of mental health care EHRs. The results also suggest that not only group education but also individual education programs that meet the needs of individual users are important. This finding is consistent with that of previous studies [19,20].

### Differences by the Job Group

Opinions were divided by the job group on organizational support for the introduction of the mental health care EHR. Among the study participants, the executive level persons thought that the institution had fully supported the introduction of EHR, but end users did not. This is because the end user needs to frequently use the mental health care EHR directly in the field.

### Differences by Age Group

The average age of participants was not high. Of the total participants, 61% (26/43) were aged between 30 and 50 years. Only 16% (7/43) of the participants were aged above 60 years. However, older age can interfere with the adaptation of the new system, combined with vague anxiety about the introduction of the new system and concern about one's own computer comprehension. The older staff had general anxiety because of their physical ability to use computers and psychological pressure. They even showed their thoughts by saying that they “[considered] quitting when they saw the system for the first time,” which is similar to a previous study showing that age is associated with satisfaction with EHR use [21,22].

### Facilitators and Barriers Relevant to the Unique Workflow of Psychiatric Health Care

One of the essential features of a mental health care EHR is that the system should accommodate its unique workflow. The psychiatry department differs greatly from other departments in terms of work processes. General tests, such as specimens and imaging tests, are performed less frequently, and questionnaires and question-based tests are performed more frequently. There are also more text-based records than other departments. As a result, EHR is more likely to serve as a greater barrier than other departments in retrieving and using psychiatric records for care. In a systematic review of barriers to EHR adoption, the author revealed that work process challenges are one of the most frequently mentioned barriers [22].

Furthermore, psychiatry has more complex privacy-related regulations than most other areas of medicine [2]. Study participants placed great emphasis on the function. Exchange and integration of medical information among psychiatric hospitals is more likely to occur when integrating federal regulations into a workflow through mental health care EHRs [2]. In fact, participants were satisfied that mental health care EHRs supported regulatory compliance. At the same time, when participants were asked to comment on how the system could be improved, they suggested to improve their function.

### Facilitators and Barriers Relevant to Training

Many participants stressed the importance of education. They mentioned several times the importance of well-executed training in implementation strategy, one of the facilitators. In the introduction of EHRs, the age of trainees is an uncorrectable factor. However, general anxiety according to age or computer literacy is an area that can be solved by personalized in-depth training. The initial barriers caused by steep learning curves or the complexity of the mental health care EHR can also be addressed with personalized training. Indeed, users were highly satisfied with scenario-based training that could occur in a clinical setting. Therefore, practical, personalized training that fits the needs of mental health care EHR users is important [23,24]. As a matter of fact, one of the participants emphasized the importance of training:

Initially I had concern on how many tabs there were to get adjusted, but after the first training session it made sense and it felt organized in a categorical way.

### Barriers That Can Be Solved by the Improvement of Mental Health Care EHR

In terms of the complexity of the system, doctors, nurses, and pharmacists had different levels of desired functions. In the case of pharmacists, for example, small hospitals do not need the complex functions used in large hospitals because of the narrow range of drugs they handle. The complex functions of EHRs, developed for tertiary hospitals, can hinder the work of small hospitals. This problem can be solved by using cloud-based EHRs. Developed in a multitenancy structure, cloud-based EHRs can provide customized services to select and use the functions required by each medical institution. Alert fatigue is one of the issues that is always mentioned when implementing the CDSS [25,26]. The problem is that first-time EHR users can have a negative perception of the system and it can interrupt the workflow, requiring continuous improvement of the CDSS for psychiatric practice [27].

### Ideas

Some pharmacists suggested a more advanced CDSS function that pairs antipsychotics with recent laboratory values and recommends appropriate medications when they issue orders. The nurses were very eager to have a stronger level of alert about legal status because it is more strict and mandatory for psychiatric hospitals to follow governmental regulations than other general hospitals. The CDSS for the mental health care EHR can be different from the CDSS for other medical departments because psychiatric patients normally have multiple medications that should be modified based on laboratory values and heterogeneous text-based medical records that should be managed in every psychiatric hospital they visit. Therefore, the CDSS for the mental health care EHR is important and difficult to develop and implement at the same time [28,29]; however, the reason the psychiatric CDSS is crucial to improving mental health and the eagerness of users for an advanced CDSS can be promoters to disseminate mental health care EHR quickly and effectively.

To use the findings of this study to effectively introduce mental health care EHRs, it is necessary to recognize that there are general difficulties in introducing EHRs, as in other medical departments. Computer literacy, alert fatigue, and steep learning curves are barriers to the introduction of any type of EHR and can be overcome by user-centered repetitive training. It is important to fully inform users about the purpose and effectiveness of EHR adoption before the introduction of EHRs and ensure that all users are well trained and use the system in advance. In addition, a function or workflow specific to psychiatry should be fully implemented in EHR.

### Limitations and Future Research

Strengths of this study include the breadth of clinical disciplines and experience possessed by the end users of the mental health care EHR whom we interviewed; users of 4 hospitals who actually introduced mental health care EHRs were interviewed to investigate in detail the barriers and facilitators in introducing EHRs. The interview methodology for the qualitative analyses also provided a greater depth of understanding about the factors that are important to end users of the mental health care EHR.

A limitation of this study is that participants were recruited from behavioral hospitals that use the same mental health care EHR system. Therefore, what the interviewees mentioned in this study may be applied only to the system. However, in this study, we tried to obtain insights that can be generalized as much as possible without being dependent on a specific system so that we could draw lessons that can be applied to psychology and EHR in general, regardless of the characteristics used by users.

Future research could collect various opinions from hospitals using different mental health care EHRs and examine whether the barriers and facilitators proposed in this study apply equally to other hospitals using different EHRs.

### Conclusions

EHRs represent a key element of health care redesign. The introduction of EHRs is also important in psychiatric hospitals because it reduces drug-related errors and medical errors by sharing medical information among medical staff and makes it easier to apply regulatory rules to the workflow. Psychiatric EHR developers and hospitals should strive to effectively disseminate EHRs by accurately recognizing end users’ perspectives on barriers and facilitators identified in this study.

## Appendix

Multimedia Appendix 1 Consolidated Criteria for Reporting Qualitative Research report.

Multimedia Appendix 2 Additional information on study hospitals and participants.

## Abbreviations

CDSS: Clinical Decision Support System
CLMA: Closed-Loop Medication Administration
CPOE: computerized physician order entry
EHR: electronic health record
HIM: Health Information Management
HITECH: Health Information Technology for Economic and Clinical Health
SNUBH: Seoul National University Bundang Hospital
UI: user interface
