# Supplementary material for: User Perspectives on Barriers and Facilitators to the Implementation of Electronic Health Records in Behavioral Hospitals: Qualitative Study
Source: JMIR Form Res. 2021 Apr 8;5(4):e18764. doi: 10.2196/18764 (PMC8063095; doi:10.2196/18764)
Supplement: Multimedia Appendix 2 [file formative_v5i4e18764_app2.docx]

Major Functions of BESTCare 2.0B (including functions of General EHR)

CPOE (Computerized Physician Order Entry)

Patient Diagnosis

Admission Order

Legal Status Order

Medication Order

Clinical Decision Support System

E-prescription/EPCS

Examination Order

Intervention Order

Dietary order

Discharge Order

Patient's Precautions

Allergy Information

Infection Information

Pregnancy/Lactate Information

Bed Sore/Fall Down Risk

Clinical Alert

EMR (Electronic Medical Record)

Physician Documentation

Psychiatric Evaluation

Medical History & Physical Examinations

Psychiatric Progress Note

Master Treatment Plan (MTP) Review

Discharge Summary

Discharge Order/Aftercare Plan

Documentation

Intake Assessment

Medication Reconciliation

Master Treatment Plan (MTP)

Problem Sheet

Master Treatment Plan (MTP) Review

Nursing Assessment

Acute Nursing Progress Note

Patient Assessments

Chemical Dependency Assessment

Psychosocial Asessment

Therapeutic Services Assessment

Group Note/Family Note

Progress Note

Medical Information Review

Chart Review

Medical Record Review

Test Result Review

Medical Record Viewer

Visit & Information Review

Journey Map

Patient Summary

Physician's Inbox

Confirm Verbal/Telephone Order

Confirm Documentation

Confirm Transcribed Document

Cosign Order/Documentation

Complete Delinquincy, Deficiency Flagged Documents

Receive E-prescription Transmission Errors

Nursing

Flow Sheet

Order Review

eMAR

Nurse's Order Entry

Check-in

Transfer/Transport

Clinical Services

Group Session Attendance & Charge Entry

Group Session Schedule Management

Group Note (includes group topic setting)

HIM

Scan/Upload Documents

Flag Delinquency & Deficiency

Monitor Signed/Confirmed Status of Physician's Inbox

Documentation Input Method Report

Pharmacy

Medication Master

Admission Instruction Master

Floor Stock Master

Order Review Queue

Order Review & Verification

Therapeutic Intervention Documentation Function

Cart Filling

Pharmacy Inventory

Interface with ADC (Automated Dispensing Cabinet)

Nutrition

Meal Issuing

Nutrition Referral Review

Nutrition Screening

Nutrition Management

Consent Forms

Patient Consent Forms

Additional function developed for mhEHR

Physician

- Exhibit_B_DR_06 : Patient's precautions (suicide, assault, elopement, etc.)

- Exhibit_B_DR_07, 08, 09, 10, 11 : Physician's note (psychiatric evaluation, psychiatric progress note, psychiatric discharge note)

- Exhibit_B_DR_12, 13, 14, 15 : Detoxification order protocols

Nursing

- Exhibit_B_NR_01 : Intake Assessment

Social Services

- Exhibit_B_SS_05, 06 : Master Treatment Plan, Problem Sheets and Review

- Exhibit_B_SS_07 : Therapy group scheduling

- Exhibit_B_SS_10 : Therapy topic and group settings

Others

- Exhibit_B_AD_02 : Outpatient visit schedule

- Indication and reminder of Legal Status

- Patient Assessment Scoring System (AIMS, CIWA-Ar, COWS, Audit-C etc.)

- Group session scheduling, attendance & Group note (developed for Therapy group scheduling, topic and group setting)

#### Supplementary Table 1 Characteristics of mental hospitals

| Facility | Location | No. of beds | mhEHR Implementation date | Interview period | (mhEHR used period) |
| --- | --- | --- | --- | --- | --- |
| Hospital A | Covina, CA | 146 | SEP 22, 2017 | OCT 31 ~ NOV 2, 2019 | 2 years  3 months |
| Hospital B | Ventura, CA | 55 | OCT 6, 2018 | NOV 4~5, 2019 | 1 year  1 month |
| Hospital C | Glendale, AZ | 100 | MAY 11, 2019 | NOV 14~16, 2019 | 6 months |
| Hospital D | Tempe, AZ | 138 | JUN 22, 2019 | NOV 18~19, 2019 | 4 months |

####

#### Supplementary Table 2 Interview Participants by facility (N=43)

| Facility | Physician | Nurse | Pharmacist | Mental health clinicians | Administrative professionals |
| --- | --- | --- | --- | --- | --- |
| Hospital A | 5 | 6 | 1 | 0 | 1 |
| Hospital B | 1 | 3 | 1 | 1 | 3 |
| Hospital C | 2 | 6 | 1 | 2 | 0 |
| Hospital D | 2 | 5 | 1 | 2 | 0 |

####

#### Supplementary table 3. Demographics according to the job group

| Category | Variables | n(%) |
| --- | --- | --- |
| **Physicians** |  |  |
| Gender | Male | 10(100) |
|  | Female | 0(0) |
| Age | 31-40 | 3(30) |
|  | 41-50 | 1(10) |
|  | 51-60 | 2(20) |
|  | 61-70 | 3(30) |
|  | ≥ 70 | 1(10) |
| Career | < 9 | 1(10) |
|  | 10-19 | 3(30) |
|  | 20-29 | 2(20) |
|  | 30-39 | 3(30) |
|  | ≥ 40 | 1(10) |
| No. of EHRs used before | 0 | 1(10) |
|  | 1 | 1(10) |
|  | 2 | 1(10) |
|  | ≥ 3 | 7(70) |
| Management Level | Director | 4(40) |
|  | End-user | 6(60) |
| Facility | Hospital A | 5(50) |
|  | Hospital B | 1(10) |
|  | Hospital C | 2(20) |
|  | Hospital D | 2(20) |

| Category | Variables | n(%) |
| --- | --- | --- |
| **Nurses** |  |  |
| Gender | Male | 5(25) |
|  | Female | 15(75) |
| Age | 31-40 | 11(55) |
|  | 41-50 | 2(10) |
|  | 51-60 | 6(30) |
|  | 61-70 | 1(5) |
| Career | < 9 | 11(55) |
|  | 10-19 | 3(15) |
|  | 20-29 | 5(25) |
|  | 30-39 | 0(0) |
|  | ≥ 40 | 1(5) |
| No. of EHRs used before | 0 | 2(10) |
|  | 1 | 3(15) |
|  | 2 | 7(35) |
|  | ≥ 3 | 8(40) |
| Management Level | Director | 3(15) |
|  | Manager/Supervisor | 10(50) |
|  | End-user | 7(35) |
| Facility | Hospital A | 6(30) |
|  | Hospital B | 3(15) |
|  | Hospital C | 6(30) |
|  | Hospital D | 5(25) |

| Category | Variables | n(%) |
| --- | --- | --- |
| **Pharmacists** |  |  |
| Gender | Male | 0(0) |
|  | Female | 4(100) |
| Age | 31-40 | 2(50) |
|  | 41-50 | 2(50) |
| Career | 10-19 | 3(75) |
|  | 20-29 | 1(25) |
| No. of EHRs used before | 0 | 2(50) |
|  | 1 | 1(25) |
|  | 2 | 2(50) |
| Management Level | Director | 4(100) |
| Facility | Hospital A | 1(25) |
|  | Hospital B | 1(25) |
|  | Hospital C | 1(25) |
|  | Hospital D | 1(25) |

| Category | Variables | n(%) |
| --- | --- | --- |
| **Mental health clinician** |  |  |
| Gender | Male | 2(40) |
|  | Female | 3(60) |
| Age | < 30 | 1(20) |
|  | 31-40 | 1(20) |
|  | 41-50 | 2(40) |
|  | 51-60 | 1(20) |
| Career | < 9 | 2(40) |
|  | 10-19 | 2(40) |
|  | 20-29 | 1(20) |
| No. of EHRs used before | 0 | 1(20) |
|  | 1 | 2(40) |
|  | 2 | 1(20) |
|  | ≥ 3 | 1(20) |
| Management Level | Director | 5(100) |
| Facility | Hospital A | 0(0) |
|  | Hospital B | 1(20) |
|  | Hospital C | 2(40) |
|  | Hospital D | 2(40) |

| Category | Variables | n(%) |
| --- | --- | --- |
| **Administrative professionals**  **(HIM, Utilization Review, Marketing)** |  |  |
| Gender | Male | 1(25) |
|  | Female | 3(75) |
| Age | 31-40 | 1(25) |
|  | 41-50 | 1(25) |
|  | 51-60 | 0(0) |
|  | 61-70 | 2(50) |
| Career | < 9 | 1(25) |
|  | 10-19 | 1(25) |
|  | 20-29 | 1(25) |
|  | 30-39 | 1(25) |
| No. of EHRs used before | 0 | 3(75) |
|  | 1 | 0(0) |
|  | 2 | 1(25) |
| Management Level | Director | 3(75) |
|  | End-user | 1(25) |
| Facility | Hospital A | 1(25) |
|  | Hospital B | 3(75) |
|  | Hospital C | 0(0) |
|  | Hospital D | 0(0) |

#### Supplementary Table 4. Barriers in using mhEHR as experienced by mental health professionals

| **Physicians** |  |
| --- | --- |
| C003: ‘Here, since it takes longer to type, obviously, and I'm not a quick typist.’  C001: ‘The barrier for me was that I'm not, as an old timer, I'm not a fast typer. So that interferes with it.’ | Computer skills |
| C001: ‘It came across as very detailed, but complicated and overwhelming. Especially for somebody like me who is not EHR experienced. I haven't had EHR before. No, I have no experience with other EHR before this one.’ | General Anxiety to EHR among staff |
| C002: ‘The first impression, honestly, was that it's going to be too much work and that it's not that easy to use.’  C005: ‘It has too many details. In the beginning when you're trying to learn it, it appeared to be too much to grasp. So in that way it was a little bit difficult. But we got used to it and then it has been helpful.’ | Complexity of mhEHR |
| C009: I think it definitely is a robust system. The thing is that a lot of it is our own doing. I don't think the (mhEHR) had anything to do with it. Forms we asked are added as we told them, but some of them need to change. Oh, for example, there is an element called ‘patient insight’. All the choices are related to poor insight, so you have to use ‘Other' and then write it. ‘That patient has fairly good insight.’’  C002: ‘**Well, I think historically though, when they came up with the templates and the content, I don't know how much input they got from the medical staff, but in my view here, they didn't get much.’**  C001: ‘You see ‘affect’. It says only 'appropriate', ‘flat', which is fine, but if you go under 'mood', 'labile' becomes under the 'mood'. It should be under 'affect'. See what I mean? So there is a discrepancy.’ | Organizational facilitating condition - Clinical contents issue |
| C002: ‘And so the channel is there, but in the process when we all launched this, the changes were very slow. ... the impression I got is that it was very slowly implemented at the time.’ | Organizational facilitating condition - Slow Modification decision making |
| C009: ‘I think the initial implementation was very good. Very good. The follow-up, not so much. As I go along and it is very difficult to communicate, to who is the one who is going to address it and what is the priority? ... I think we make a request to the hospital identified person and we don't know then what happens.’ | Organizational facilitating condition - Poor Modification Request Management |
| C001: ‘The only downside is with the EHR, which you probably know, is when you are interviewing with the patient, you have to make sure you spend very little eye-contact with the EHR. What I do is I have it there. I review certain things but I maintain. I refer to it when I have to check something or let's say I want to check the vital signs, I want to check their withdrawal score, I want to check their labs. I do that and I'll tell them, but at least 70% of the time you should maintain that eye contact.’ | Potential drawback in Patient-physician relationship |
| **Nurses** |  |
| B022: ‘We have some nurses that aren't really used to working on computers. They didn't have to before for this job really. That was the biggest challenge.’  B015: ‘Yes. To be completely honest it varies. It really varies depending on a person's familiarity with computers in general, because some people are very comfortable with it, but, some people that don't use it every day, it's a little harder for them. It's just because they're not used to using a computer every day.’  B006: ‘Well, there were some nurses who weren't used to the technology, so that was difficult for them.’ | Low Computer Literacy |
| B031: ‘The first day I came, I thought maybe I'm going to put out my resignation letter. I didn't know where this was leading me to.’  B001: ‘I think there was some fear that’s more just personal. We've been paper here for a really long time, so, and there were a lot of employees that did not have the experience with an electronic health record. So there were some people that definitely were more challenging for.’  B031: ‘My Director called me saying "(Name), it's not only you. Everybody is doing this back to back. I said, "I'm good. I'm flunking up. I don't even know where to start.”’ | General anxiety to EHR among staff |
| B026: ‘For the nursing piece, there's quite a bit to navigate through finding the medical care plans, finding the Order Entry. The navigating for nursing is by far the hardest in the building and the most in-depth.’ | Complexity of mhEHR |
| B006: ‘It's just that, to learn so many new things all at once was the hard part, I think.’ | Steep Learning curve |
| B017: ‘They're constantly getting the alerts and sometimes the nurses will turn that feature off to not have to keep getting those alerts.’ | Alert Fatigue - New Order Alert |
| B024: ‘I think it may have been a different story had we never had it. But to take 200 nurses and then retrain them, it's been difficult.’  B026: *We had the scanning features. We had the (CLMA) alerts. It's really just fine tuning so that they feel comfortable with it.* | Resistance due to experience with Legacy system |
| B005: ‘This wouldn't really be (mhEHR), but policy-wise as well, helping the hospitals with that too. Because we had to go through each individual policy and document change things. But that might be more of a (corporate) issue. Policies and procedures had to change all from paper documentation to reflect electronic. Yeah. So that's more so, I think probably (corporate) aspect.’ | Organizational facilitating conditions - Revise policies to reflect EHR |
| **Pharmacists** |  |
| B027: If you're in a larger hospital where there's a lot of staff, this is appropriate. But where we don't have as much staff, I think, and we're a smaller hospital, I think all of the different features, it's a little too much and overwhelming for everyone.  B027: ‘I don't think the system has a problem. I think it just requires more manpower than what we have available to us. I feel like if we were a pharmacy that was running, and we have two pharmacists on all the time, and we have two techs, and we're running a bigger pharmacy, I think it's an appropriate system, because you have the time to go back and forth and look up all the information. Where we're a one pharmacist, one tech team, and so having to go around to all these different modules, and do all these different things to find one piece of information is not helpful, if that makes sense.’ | Complexity of mhEHR - Too many features for a small pharmacy |
| B010: ‘What came up was "high dose over recommended max," but there's so many alerts that I feel as if everyone's going through decision fatigue, because everyone's just like, "bypass, bypass,". Because there's so many.  B020: ‘Then when you verify an order, the drug alerts that come up, it tells you the major ones, the minor ones, there's way too many. Definitely an alert fatigue. Then alerts where there's a duplicate therapy so the patient might be on two NSAID medications, anti-inflammatories, that's a very minor alert. If you're verifying a few meds, you might have this entire screen filled with alerts, and that might be all the way at the bottom. | Alert Fatigue - CDSS |
| B020: ‘Our old system, everything was on one screen. The doctors were entering orders on one screen. We were verifying on the same screen. It was all done on the same screen. There wasn't the Order Entry, the review, the verify. There's three screens now, where everything was just done on one screen. It wasn't all of this information.’  B020: ‘Previous to (the mhEHR), we did have an electronic MAR. We were using a system there, so the comparison between the two, this was a lot more detailed, more information on the screen.’  B027: ‘Because they're used to (Legacy system). We were able to verify, fix things, do things very quickly, and now I have to go from ‘Order Review and Verify’, then I have to go over to the ‘Order List’, and then switch the date and do this and do … whereas before it was just different tabs we could just click, click, click, click, click all on the same main, one screen.’ | Resistance due to experience with Legacy system |
| **Mental Health Clinicians** |  |
| B021: ‘My team has a large demographic of different ages and so those who were not as comfortable or as quick as typers had a little bit of a challenge getting used to typing so much.’ | Low computer literacy |

#### Supplementary Table 5. Facilitators in using mhEHR as experienced by mental health professionals

| **Physicians** |  |
| --- | --- |
| C001: ‘(mhEHR) is more comprehensive and more relevant to psychiatry. With (other EHRs), relevance to psychiatry is not high. They have a little bit of a section of psychiatry. This is more relevant, and I find it comprehensive, easy to use, and navigate it very well.’  C005: ‘I go to (OOOO hospital), (OOOOO Medical Center), and I have an EHR in my office. Compared to those, this seems to be more comprehensive.’  C004: ‘I go to multiple hospitals and each hospital has a different one. Most of them are just medical and we have to apply and adapt to it. This system is very psychiatric friendly.’ | Comprehensive Mental Health centered EHR |
| C006: ‘The nice thing about (mhEHR) is you can be forced to not have any deficiencies because you can't move on, you can't sign your note unless you've addressed everything.’ | Regulatory compliance |
| C004: ‘Ordering is pretty self-explanatory. … Overall it's well presented. I think presentation-wise it is clear.’  C001: ‘To give you another feedback, we do have medical students here. They are young, they are fast typers, they have exposure to several EHRs. It takes them only probably a day or two to get acclimated, and they love it.’ | Easy to use |
| C004: ‘ I feel it’s much more efficient. I feel super comfortable with the system. I understand it, everything where it’s at. | Improved efficiency |
| C004: ‘I think initially there were a lot of tabs that made some sort of concern on how many tabs there were, to kind of get adjusted. But after the first training session it actually made sense and it felt organized in a categorical way.’  C001: ‘What I do now, the initial Psychiatric evaluation, I use a hybrid. What I mean is I dictate the narratives, (Dictation company) sends it to my (mhEHR) inbox. I go over there and complete it. For the old timers, those who don’t type fast, it works very well.’ | Good usability |
| C004: ‘It’s very good to have (the mhEHR) where everything is easily accessible, especially with patients who have readmissions, the information is right there.’  C001: ‘Well, the main value really it was very good to know the medication that the patient's taking right away and admission reports. The other people reports, easy just with a click. I can get into those things. To navigate through the charts from one topic to another or one area to another. It's very easy to do that.’ | Easy access to patient information |
| C005: Medication errors are definitely going to go down. In this way everything is in print. So everything is concrete so that if it is a mistake, if the doctor's ordering in different ways, the nurse can visualize and then say, “Hey, this is the wrong way”, communicate with the physician because she can see the order very clearly. It definitely improves the patient's safety.  C004: I think patient safety has improved because the orders are immediately inputted and the safety nets that we have are immediately notified. For example, our pharmacy can notify about changing medication adjustments as opposed to a time lapse once medications were ordered which can happen with paper.  C005: Even though initially there has been a lot of criticism and scary feelings and all that I think all those things have subsided and it has definitely improved the quality of care. Not only the patient's safety and then the quality of care and documentation.  C003: And if people(physicians) are putting in their own orders, it improves patient care since less mistakes are made that way. Instead of doing telephone orders, when the nurse hears something else, they order something else. So it cuts down on medications mistakes.  C004: ‘It(CDSS) gives that extra layer of protection for the patient.’  C001: I think the (CDSS) alerts are fine. The more the better. It’s better not to miss. Yes. | Improved patient safety & quality of care |
| C001: ‘I think the communication is better because it is expedient. Of course, what determines good communication is the content that's in the mhEHR.’ | Enhanced interprofessional communication |
| C004: ‘The first training experience was actually very, very good. I felt very comfortable using the system.’  C001: The one-to-one instructions were much better. So I had to spend about two hours. One hour, two sessions, to get acclimated and then I started using it. Now it's fine. | Well-executed training |
| C005: ‘I think they have been very kind and they have been very patient. And that was very helpful. Compared to the other hospitals where I work, we didn't have this much support. And they have taken a good amount of time for the implementation.’ | Sufficient time |
| C004: ‘I haven't had any comments or negative comments in regards to us turning into our EHR. On the contrary, letting the patient know that “Right now, I'm reviewing your medication from the computer” seems like it makes them feel involved in the treatment at that time as opposed to the paper.’ | Positive Physician-patient relationship |
| **Nurses** |  |
| B001: ‘(mhEHR) is drastically different. I think it fits well for what we do, workflow-wise. I have no issues using it, training on it.’ | Mental Health centered EHR |
| B001: *‘*I tell people this when I'm training them, if you can use your smartphone, you can use (the mhEHR). It's that simple. I tell people it's one of the easiest systems I have used. So that would make it in itself one of the best systems. And I have had a few nurses that have worked at other hospitals and they too have said that this is one of the easiest systems that they have used. Overall it's very easy to follow. It's very easy to figure out and you can solve it on your own. So, yes, it's definitely efficient, effective, and easy.’  B001: ‘And when I first started to see it, it became clear to me that it was going to be pretty easy. So very user friendly and easy to follow. I liked it. I made the correlation between a smartphone with the apps. So, my brain got it.’ | Easy to use |
| B019: ‘Overall it's amazing. I think timewise, it has helped us to work within time, before you'd work in off a longer period of time because you have to do the paper charting. Timewise it has helped us because everything is right there.’  B005: ‘I think navigation-wise it's good. I like that you can view two screens at the same time, because that really helps. If I'm talking to a patient about meds, I can have my documentation open, but also looking at the meds. We like that and a lot of the nurses like that.’ | Improved efficiency |
| B006: ‘It actually makes my job so much easier because I can access the information from whatever location I'm at, as opposed to going to the units and pulling out the charts. And it's easier for me to get the information at the same time someone's looking into the chart, because otherwise I have to wait to see when it's available.’ | Easy access to patient information |
| B031: ‘If anything happened, even though if you are not there, your colleague or your boss can easily go there to see what you have done so far, so they would cover you and talk for you. They might not require you anymore, because they have seen what they're looking for.’ | Easy to track information, work progress |
| B001: ‘Especially with the alerts just for medication alone. Because before on paper, we'd have to wait anywhere from 20 to maybe two, three hours when pharmacy would get the information, enter it and realize, Oh, that patient has an allergy or these two medications are contraindicated. That system tells you right then and there. So that's helpful.’  B001: ‘I know our medication errors have gone down drastically, which is good.’  B021: ‘Some nurses are not familiar with what the medications are, so I think (the CDSS alerts) are a good thing.’ | Improved Patient Safety |
| B019: ‘I think communication is better with (mhEHR) because there is continuity of care, communication amongst the team, the different disciplines. I think (mhEHR) has really put us to another level unlike before. I can see if I'm working on that patient and the counselor is working on that patient, I'm able to read the note what the patient did in the group. So I think it's really amazing. | Enhanced interprofessional communication |
| B032: ‘I think the people that came out and trained us last time were really helpful. I really liked them. I felt like they were always there when we needed them. So the training was for, in my opinion, really, really good. I don't think that that could have been much better.’  B031: ‘When I attended the third time, I met one of the senior trainers. So I called him, I said, "You know what? This, your (mhEHR) is going to send me out of job." He started laughing. He said, "(Name), I know you can do it." Then he said, "Sit down. When you come to the office, what do you first do?" I said, "I look at my patient.". "When you look at your patient, what do you do?" I said, "I look at their chart". He says “It is the same thing here. You come, you look at their charts, you see...” And he started showing me. That was the end. The third time, I got it. The first time, I was totally lost.’  B001: ‘As it started with the training process with all of (the trainers) here, it felt very supportive. All questions were asked in a timely fashion.  B004: ‘I think what would really help a new hospital is expanding on the current training method. What you're doing with the scenarios, provide more scenarios.’ | Well-executed training |
| B019: I would have a recommendation, maybe some more time, which they did give us. I think the trainers we had really did a good job. They gave us time, they weren't like hurrying us, no. So I would put emphasis on that. | Sufficient time |
| B001: ‘So it's almost exactly what we were doing on paper. Just a computer form, quicker, better, nicer.’ | Implementation planning |
| **Pharmacists** |  |
| B010: It(CDSS) definitely enhances patient care… It's better than having paper. | Improved patient safety & quality of care |
| B010: ‘I think for me (the communication is) more enhanced, because if I compare it to just the one site I was at, more nurses are talking to me than when I was there.’  B010: ‘So let's say there's an antipsychotic and it needs certain labs and I don't see them ordered, I'll send a consultation or the documentation to the doctor. I'll see that they actually change the orders within the same day. Then occasionally they'll call, and they'll be like, "Hey, what's going on with this?" And it's the response to my consultation.’ | Enhanced interprofessional communication |
| **Mental health clinicians** |  |
| B012: ‘All the clinical forms and everything that we need to do, treatment plans, all that, it flows nicely.’ | Mental health-centered EHR |
| B009: ‘We go into this person's treatment plan, I go around and I pass my laptop around to each person and they all sign.’ | Support multidisciplinary documentation |
| B033: ‘Most of it was intuitive. The training was good and then once we practiced with it a little, we were able to figure it out. So it came on really quick.’ | Easy to use |
| B021: ‘After it launched, I was very excited. It was quick and fast. Going from printing out over 200 documents a day and using over 200 labels a day, running out of pen ink, hand cramps. So I was excited that it was electronic because things were done smoother.’  B033: ‘The main thing is that the Group notes system made everything much quicker … that was a concept that was really easy for people to understand.’  B033: ‘What we have now is efficient and it's cut a lot of time out because we used to have to sign all the notes then go and put them all in the charts. That was terrible. It just took forever.’ | Improved efficiency |
| B021: ‘The favorite menu for me is my favorite. Because I can really just save the documents that I regularly use and search from there. The quick mhEHR shortcuts. Those shortcuts make a huge, huge difference.’ | Personalization option |
| B025: ‘I think hands down (mhEHR) is new age when it comes to user friendly, as far as the systems. The systems I've used, they feel archaic. You can tell that even though they may be new, it hasn't been revamped, implemented with new ideas in some time. So I think (mhEHR) is phenomenal in that regard.’ | Good usability |
| B021: ‘It’s alive. We can watch people do their work. Everything's accurate. So you can see when someone's doing something and communicate if there is a potential error.’  B033: ‘It makes it(reviewing) very, very easy. I can count all the notes easily from the documentation tab and I can very easily see when assessments are done and completed. So that really helps me.’ | Easier tracking of work progress |
| B033: ‘They got the retrieve button for (the patient’s) problem … so that makes it much easier. So the problem will populate itself and then another retrieve button for the topic.’ | Useful  auto-retrievals |
| B021: ‘As far as communication as a whole, I found that we've had social workers come up to us in our department, "Wow, your notes are so detailed," and "Oh, your notes are really descriptive with your team," and it's great that they're really reviewing the chart and taking time to read other's documentation.’ | Enhanced interprofessional communication |
| B021: ‘You can really go back and see like this patient told me X, Y, Z. But just yesterday he said something different, because it's right here. So it helps our facilitation of therapy and really building rapport, because we have it all at our fingertips. We kind of have a background of going in with our patients of all the collateral information. So yes, I think it's helped.’ | Easy access to patient information,  Improved quality of care |
| B021: ‘We had such great support from (mhEHR) team and from the staff that came out to train us.’  B012: ‘I thought that the rollout was really good. I think it was very thorough in teaching all of us what we needed to know.’ | Well-executed training |
| B021: ‘It was a smooth transition. We did that soft launch and then we were talking about it for a month or so before. I felt prepared and open.’ | Implementation planning |
| **Administrative professionals** |  |
| B013: ‘I'm in charge of all the joint commission and CMS surveys, and (the mhEHR) has everything in there that a surveyor would want to see. I got to give it to them, they did a good job form-wise, to make sure everything was there.’ | Ensured regulatory compliance |
| B013: ‘All the nursing staff, we tried to help them set up their favorites and all that so it would be ready to go.’ | Personalization option |
| B007: ‘And I find it very easy. Now that I do it all the time, it's very quick. "Oh I need to look at the Intake Assessment, physician's Psych Evaluation, the Discharge Aftercare Summary." And then I just double click on it, and I open it, and there it is.’  B007: ‘(mhEHR) has really helped me because it used to be I could never get information. I'd have to go pull the patient's chart, I'd have to go to the unit, I'd have to go to Medical Records and try. So it wasn't until we got the (mhEHR) that I could even look this up. Before, we weren't capturing that. It was captured on the paper chart, but I couldn't find it easily at all.’ | Easy access to patient information |
| B013: ‘Usually when we have a problem and you get it done pretty quick, and no EHR is perfect. I know that. But as long as when we need something changed, it's fairly easy to change it. I know I've asked (Corporate support) to switch a couple things around and he can do it himself, which is nice.’ | Flexibility of mhEHR |
| B013: ‘I think we had an advantage because we were closed. Trying to learn this and work and implement and start it while you're taking care of patients and you're converting from paper to computer, I think would have been a little harder. So that was better for us. I think that was really helpful.’  B013: ‘Easier than somebody that's full and super busy and you're trying to learn this whole new (mhEHR). To do your day to day job at the same time I think would be difficult.’ | Sufficient time |

#### Supplementary Table 6. Ideas for future enhancements in behavioral EHR

| B013: ‘Maybe by suspending some kind of function to where it's not going to let you move forward until this is addressed. A pop up screen that offers, “not addressed” or “addressed”. So, it can change the legal status to show that the user has not taken care of this order yet. If it was not addressed, let's say by either discipline, doctor or nurse, hold ended one minute ago, boom, a pop up should appear. Showing it that the user needs to obtain legal status or order needs to be written. It's day two now and it still says pending, because somebody didn't address it. I think that would be helpful.’  B026: ‘If somebody is on a one-to-one, our policy is it has to be renewed every eight hours, Q8 hours. Then our blocked beds are every 24 hours. Probably an hour before. 30 minutes before just to say, "Hey, don't forget to renew your one-to-one".’  B011: ‘Legals. So hold is up and probably nurses need to be bombarding like, ‘Address hold’, ‘Address hold’, ‘Address hold’. So, all doctors. You're doctor access the system and it would just pop up, ‘You need to address hold’, ‘You need to address hold’. | Alerts to address Legal status |
| --- | --- |
| B010: ‘So when you have certain medications that are ordered, certain antipsychotics, I would think it would be helpful if ones would pop up and then the doctor could select, ‘Depakote, liver enzyme test’ where they don't have to actually remember it and it just pops up, and then they just select which ones.’*‘* | Auto-recommendation of labs when issuing antipsychotics |
| B033: ‘If the tablet had (the mhEHR) that I could go to them for the assessment part, that would make it easier. Because then I could tell them I'm just going to do an assessment real quick and check the boxes. And at the end there's one sentence that we write that would make it easier cause right now I'm doing that all on a piece of paper and then I'm going down to my computer and putting it in. It's really quick. It's quicker than it was before, but a tablet, I wouldn't even have to print out any pages. I could do it, save, then go back and put the narrative in. That's just a very small part, which we might even be able to figure out. Yeah, that'd be cool. Really nice for assessments.’  B005: ‘In code situations it would help too, for documentation. Especially our code blues, if we're having to do CPR or even just administering meds, it would help with documentation, rather than having somebody sit at a computer and entering that after.’  B003: ‘If you were to do something with a tablet or something mobile, those round sheets would be a good idea. They are designed for a psych hospital based on state surveys to have certain things. The round sheet tells you what time, where the patients are, and what they are doing, basically every 15 minutes. So it is coded pretty quickly. Sleeping is a certain number. Walking, pacing is a certain code. The way I'm imagining it is there's location, current behavior and time. Maybe they can click like ‘R’ for the restroom.’  C006: ‘I think if we had a tablet and the mental health worker doing the rounds does it, it's entered in the system right away versus you're writing it on paper and then having to put it in later. So I think it's much more efficient and less chance for errors to do it with a tablet right there.’  B029: ‘I think tablets would be good in the Intake rooms when we're doing all the consents for the meds, because we have them just hand signing everything. I think it actually would be great in the Intake. I think that would speed things up and help out. Absolutely, it would. 100%.’  B013: ‘Because every 15 minutes we have a sheet of paper that has a thousand check boxes and you check where they were, what they were doing. Every 15 minutes. Someday if we can come up with a form and have it all on a tablet.’ | Use of Tablets in Group therapy sessions/Code situations/Round sheet/ Consents |

Medical Record Implementation in Mental Health Care: A Case Study

Interview guideline for interview research

| **Preparation for Interview**  Prepare a room or a space for interview  Explain the purpose of the interview precisely to the participant.  Address terms of confidentiality  Explain the process and format of the interview  Tell participants “Feel free to Ask if they have any questions”  **Topics in Questions**  Behaviors - about what the participant has done or is doing  Opinions - about what the participant thinks about a topic  Feelings - about what the participant’s feeling about a topic  Knowledge - collect facts about a topic  Background/demographics - get background information, such as age, education, occupation, and etc.  **Sequence of Questions**  Get the participant involved in the interview as soon as possible.  Before asking about controversial matters , first ask about some facts.  Ask the participant any idea he or she wants to add for the last question  **Wording of Questions**  Use open-ended and neutral questions  Ask clear questions.  **Conducting Interview**  Ask one question at a time.  Attempt to remain as neutral as possible.  Provide transition between major topics.  **Immediately After Interview**  Tell interviewees how to get in touch with researchers later if they want to  Verify if the audio recorder worked well.  Adapted from Field Guide to Consulting and Organizational Development: A Collaborative and Systems Approach to Performance, Change and Learning Paperback – January 1, 2006 by Carter McNamara |
| --- |

**Themes identified**

| Category | Theme | n(%) |
| --- | --- | --- |
| **Barriers** | Computer Literacy | 12(27) |
|  | General anxiety to EHRs among Staff | 18(41) |
|  | Complexity of mhEHR | 23(53) |
|  | Steep Learning Curve | 23(53) |
|  | Alert Fatigue | 14(32) |
|  | Organizational facilitating condition | 12(27) |
|  | Resistance due to experience with Legacy system | 8(18) |
| **Facilitators** |  |  |
| Mental health centered EHR | EHR tailored to behavioral workflow | 19(44) |
|  | Support multidisciplinary documentations | 2(4) |
|  | Support regulatory compliance | 4(9) |
| Well-designed EHR system | Easy to use | 18(41) |
|  | Improved productivity | 16(37) |
|  | Good usability | 20(46) |
|  | Flexibility of forms | 2(4) |
| Advantages of using an EHR | Easy access to patient information | 12(27) |
|  | Improved patient safety and quality of care | 5(11) |
|  | Enhanced inter professional communication | 18(41) |
| Implementation Strategy | Well executed training | 20(46) |
|  | Sufficient implementation time | 4(9) |
|  | Implementation planning | 13(30) |
| **Ideas** | Alerts to address Legal status | 7(16) |
|  | Auto-recommendation of laboratory exams for antipsychotics | 2(4) |
|  | Use of Tablet PCs | 16(37) |
